# Hypertonic dextrose prolotherapy in osteoarthritis: mechanisms, efficacy, and future research directions

**DOI:** 10.3389/fendo.2025.1602727

**Published:** 2025-08-04

**Authors:** Kai Huang, Haili Cai

**Affiliations:** ^1^ Department of Orthopaedics, Tongde Hospital of Zhejiang Province, Hangzhou, China; ^2^ Department of Ultrasound, The 903rd Hospital of People's Liberation Army (PLA), Hangzhou, China

**Keywords:** osteoarthritis (OA), dextrose prolotherapy (DPT), hyaluronic acid (HA), platelet-rich plasma (PRP), physiotherapy

## Abstract

Osteoarthritis (OA) is a degenerative joint disease that causes pain, stiffness, and reduced mobility, significantly affecting the quality of life for millions globally. Current treatments focus on symptom management, with limited efficacy in addressing the underlying disease progression. Hypertonic dextrose prolotherapy (DPT) has emerged as a potential regenerative treatment, utilizing dextrose injections to stimulate the body’s natural healing mechanisms by promoting tissue regeneration, strengthening ligaments, and improving joint function. Recent clinical studies, including the latest research findings, have shown that prolotherapy offers significant pain relief and functional improvement in knee OA, often outperforming other treatments like hyaluronic acid (HA) and corticosteroid injections. Despite promising results, the efficacy of prolotherapy varies across studies due to differences in protocols and study designs. Challenges remain, including lack of standardization, methodological bias, and short-term follow-up. Future research with rigorous designs and long-term follow-ups is necessary to establish prolotherapy’s role in OA management, ensuring a more comprehensive understanding of its therapeutic potential.

## Introduction

Osteoarthritis (OA) is a degenerative joint disease characterized by progressive cartilage deterioration, resulting in pain, stiffness, and reduced mobility. The gradual loss of cartilage causes bones to rub against each other, further impairing joint function and exacerbating discomfort. Globally, OA significantly contributes to chronic pain, affecting more than 600 million people, particularly older adults (1). A systematic review involving 34 studies conducted over 25 years among populations aged ≥15 years in South Asia, East Asia, the Pacific, and Sub-Saharan Africa reported a pooled prevalence of 16.05%, indicating approximately one in six individuals suffer from OA, despite notable heterogeneity across studies (2). While the knee, hip, and hand joints are most frequently affected, OA can develop in any joint. Additionally, OA-related physical impairments often lead to psychological distress, including depression and social isolation (3). Beyond the individual burden, OA poses a growing public health challenge. According to the Global Burden of Disease Study, approximately 595 million people worldwide were living with OA in 2020, and this number is projected to rise to over 1.1 billion by 2050. OA is now ranked as the seventh leading cause of years lived with disability in individuals aged over 70 years. The socioeconomic impact is substantial, with high direct healthcare costs and productivity loss, particularly in low- and middle-income countries where the disease burden is increasing rapidly (1). Despite growing insights into OA pathogenesis—including genetic, mechanical, inflammatory, and metabolic contributors—current therapies primarily focus on symptom control rather than disease modification.

Currently, OA treatment focuses primarily on symptom management rather than addressing the disease’s underlying progression. Common therapies include nonsteroidal anti-inflammatory drugs (NSAIDs) and corticosteroids, which alleviate pain and inflammation but fail to repair damaged cartilage (4–6). Long-term use of these medications can lead to serious side effects such as gastrointestinal and cardiovascular issues. Intra-articular injections of hyaluronic acid (HA) aim to restore joint lubrication, but their efficacy is limited, and they do not reverse cartilage damage (7, 8). Similarly, corticosteroid injections provide short-term relief but may accelerate cartilage degeneration and joint infection with repeated use (9, 10). When conservative treatments fail, surgical options like joint replacement may be considered, but these procedures come with significant risks, including infection and long recovery periods (11). These limitations have sparked interest in exploring regenerative medicine as a potential alternative.

Hypertonic dextrose prolotherapy (DPT) has emerged as a promising regenerative treatment for OA. This technique involves injecting a solution of dextrose (ranging from 12.5% to 25%) into the affected joint or surrounding tissues. The injected solution induces a localized inflammatory response, stimulating the body’s natural healing process and promoting tissue regeneration. While the exact mechanisms remain unclear, it is believed that prolotherapy activates growth factors and cytokines, encouraging the formation of new connective tissue, strengthening ligaments and tendons, and stabilizing the joint (12). This regenerative process may improve joint function, reduce pain, and potentially slow OA progression (13). Developed in the 1950s by Dr. George Hackett for musculoskeletal pain, DPT has since been applied to treat various conditions, including OA. Early studies on its efficacy, particularly in knee OA (KOA), have shown promising results, with significant reductions in pain and improved function (14). However, the evidence supporting DPT’s use in OA is still evolving. Some studies report positive outcomes (15, 16), while others show minimal or no benefit compared to placebo or other treatments like HA and corticosteroids (17). This variability may stem from differences in study design, treatment protocols, and patient populations. Nonetheless, DPT’s potential to stimulate tissue repair presents an exciting area for further research, particularly given the limitations of current OA therapies.

## Literature selection criteria

Given the narrative nature of this review, we conducted a focused literature search to ensure the inclusion of high-quality and relevant studies. We systematically searched databases including PubMed, Web of Science, and Embase for English-language articles published between January 2008 and May 2025. Search terms included combinations of “dextrose prolotherapy,” “hypertonic dextrose,” “knee osteoarthritis,” “platelet-rich plasma,” “hyaluronic acid,” “physiotherapy,” and “intra-articular injection.” Priority was given to randomized controlled trials (RCTs), meta-analyses, and systematic reviews involving human participants. Studies were included if they investigated the clinical efficacy, biological mechanisms, or safety profile of DPT in comparison to standard treatments for knee osteoarthritis. We excluded case reports, editorials, non-peer-reviewed articles, and studies focusing on other joints or unrelated conditions. The selected literature was evaluated for methodological quality and relevance to the core themes of this review.

## Biological mechanisms of DPT in OA

OA is a multifaceted joint disorder characterized by the degeneration of articular cartilage, alterations in the extracellular matrix (ECM), and remodeling of the subchondral bone. In OA, the articular cartilage undergoes structural and biochemical transformations. Inflammatory cytokines and mechanical stress disrupt the balance between cartilage matrix synthesis and degradation, leading to the breakdown of collagen fibers and proteoglycans. This degradation impairs the cartilage’s ability to retain water, resulting in reduced elasticity and shock-absorbing capacity. Consequently, the cartilage becomes thinner and less resilient, contributing to joint pain and stiffness. Beneath the cartilage lies the subchondral bone, which also undergoes significant changes in OA. Early in the disease, increased bone turnover leads to a thinner, more porous subchondral bone plate and deteriorated trabecular bone. As OA progresses, the subchondral bone becomes denser and more sclerotic. These alterations affect the bone’s mechanical properties, potentially exacerbating cartilage degradation and contributing to pain (18). Understanding these interconnected changes in cartilage and subchondral bone is crucial for developing targeted therapeutic strategies aimed at preserving joint integrity and function in OA patients.

**Figure 1:**
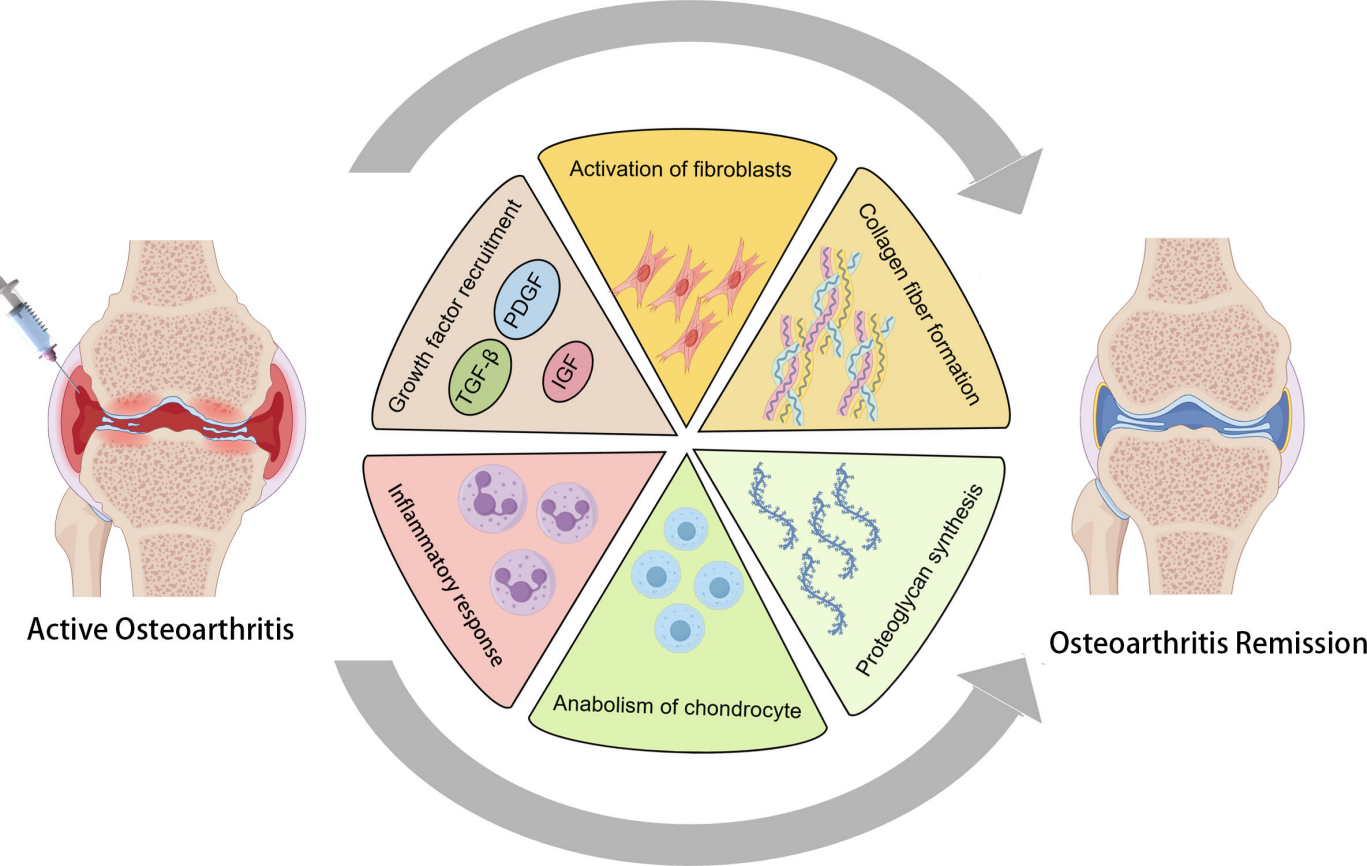
The mechanism of action of dextrose prolotherapy (DPT) in treating osteoarthritis (OA) involves the induction of a controlled inflammatory response through the injection of dextrose. This localized inflammation activates the recruitment of key growth factors, including TGF-β, PDGF, and IGF, which play crucial roles in tissue repair. The process simultaneously activates fibroblasts, stimulating collagen fiber formation, while also promoting the anabolic stimulation of chondrocytes and the synthesis of proteoglycans. Together, these actions contribute to the resolution of OA symptoms and facilitate tissue regeneration.

DPT is a regenerative injection technique aimed at promoting tissue repair and alleviating pain in musculoskeletal disorders. The treatment involves injecting an irritant solution, commonly dextrose, into areas with damaged tendons, ligaments, or joints to stimulate the body’s natural healing processes. Dextrose, a simple sugar, serves as the primary proliferant in prolotherapy. When injected, it induces a controlled inflammatory response at the targeted site. This localized inflammation recruits growth factors and cellular mediators essential for tissue healing, thereby enhancing the repair of injured connective tissues (19). The intentional inflammatory reaction triggered by dextrose injection leads to the activation of fibroblasts, the cells responsible for synthesizing collagen. This process results in the deposition of collagen fibers, which are fundamental components of the ECM, contributing to the strengthening and stabilization of the affected tissues (20). Beyond collagen synthesis, DPT stimulates the release of various growth factors, such as platelet-derived growth factor (PDGF), insulin-like growth factor (IGF) and transforming growth factor-beta (TGF-β). These growth factors play pivotal roles in cell proliferation, angiogenesis, and ECM remodeling, collectively facilitating the regeneration of damaged tissues and improving overall joint function (21). In summary, DPT utilizes dextrose injections to deliberately induce a healing cascade characterized by inflammation, collagen deposition, and growth factor activation. These mechanisms collectively enhance tissue repair and regeneration, offering potential relief for individuals suffering from chronic musculoskeletal conditions (**Figure 1**).

## Clinical evidence supporting DPT for knee OA

Several studies have evaluated DPT’s impact on pain and function in OA patients. A randomized controlled trial compared the efficacy of DPT at 5%, 10%, and 20% concentrations in knee OA patients. All DPT groups showed significant improvements in pain, function, and joint flexibility versus exercise-only controls. While no significant differences were found among the DPT groups, the 20% concentration demonstrated the greatest improvement in Visual Analogue Scale (VAS) and Western Ontario and McMaster Universities Osteoarthritis Index (WOMAC) scores and knee flexion. The study concludes that 20% DPT is the most effective and recommends its use for KOA, though long-term and placebo-controlled trials are still needed (22). A meta-analysis included 14 randomized controlled trials with a total of 978 patients and evaluated the effectiveness, compliance, and safety of DPT for KOA. The results demonstrated that DPT significantly reduced pain and improved physical function and quality of life compared to placebo. The analysis also found that a greater number of dextrose injections and longer follow-up duration were significantly associated with better outcomes, suggesting dose- and time-dependent therapeutic effects. Combined intra-articular and extra-articular injections showed superior pain relief compared to intra-articular injections alone. Moreover, dropout rates were lower in the DPT group, indicating better treatment compliance, and no significant increase in adverse events was reported. Although the study supports the potential of DPT as an effective and safe alternative treatment for KOA, considerable heterogeneity and risk of bias among the included trials warrant cautious interpretation (23).

## Comparative effectiveness of DPT and other therapies

### DPT vs. normal saline

A randomized controlled trial conducted in Hong Kong (24) compared intra-articular DPT with NS injections for KOA. The results revealed significant benefits for the DPT group, including a substantial reduction in pain, improved function, and enhanced quality of life. Notable improvements were observed in the WOMAC pain, function, and composite scores, as well as in VAS pain intensity and EuroQol-5D scores. These positive effects persisted for up to 52 weeks. No adverse events related to the injections were reported, and patient satisfaction was high. In contrast, although the NS group also showed some improvement, these changes were less pronounced, suggesting that DPT is a more effective, safe, and cost-efficient treatment for KOA, particularly for patients who do not respond to conventional therapies.

### DPT vs. HA

A 2019 review compared DPT with HA injections in KOA. Both treatments effectively reduced pain and improved function, with DPT showing comparable, if not superior, outcomes (25). Waluyo et al. (26) investigate the effects of DPT compared to HA injections in treating KOA. DPT was found to significantly reduce urinary C-terminal telopeptide of type II collagen (uCTX-II), a biomarker of cartilage breakdown, while showing more substantial pain relief and functional improvement than HA. Both treatments improved pain and function, as measured by WOMAC and NRS scores, but DPT outperformed HA in reducing cartilage degradation biomarkers. These findings suggest DPT as a promising, cost-effective alternative for OA management, particularly in resource-limited settings. On the contrary, Hosseini et al. (27) compares the efficacy of periarticular hypertonic DPT and intraarticular HA injections in treating KOA. A total of 104 KOA patients were randomly assigned to receive either DPT or HA injections. After three injection sessions, both treatments significantly improved pain levels and knee function, as measured by VAS and WOMAC scores. However, HA injections were found to provide more significant symptom relief than DPT, suggesting that HA may be the more effective option for managing KOA symptoms. These findings support HA as a preferable non-invasive treatment compared to DPT. Based on the comparison between DPT and HA for treating KOA, the recent study (28) found that DPT, when combined with physical therapy (PT), resulted in more significant pain reduction and functional improvement than HA+DPT. Although HA+DPT also showed effectiveness, DPT+PT was superior in achieving long-term benefits.

### DPT vs. platelet-rich plasma

A randomized, double-blind clinical trial by Rahimzadeh et al. (29) evaluated the efficacy of intra-articular PRP and DPT in patients with KOA. The study demonstrated that both interventions significantly alleviated pain and improved knee function. However, PRP produced more pronounced and sustained benefits over a six-month period, suggesting it may be the more effective option for managing KOA. Nonetheless, the higher cost and technical demands associated with PRP may limit its widespread adoption in routine clinical practice. In contrast, a comprehensive network meta-analysis by Liao et al. (28) analyzed data from 80 randomized controlled trials involving over 6,900 patients and offered a broader perspective by evaluating both monotherapies and combination regimens involving physical therapy (PT). The findings revealed that when combined with PT, both PRP and DPT provided superior outcomes compared to their standalone counterparts. Notably, the DPT+PT combination emerged as the most effective for pain reduction and global functional restoration, especially in immediate and short-term follow-up periods. While PRP+PT also demonstrated substantial efficacy—particularly in enhancing walking capacity—it was slightly less effective than DPT+PT in relieving pain during the early phases of treatment. The discrepancy between these two studies can be attributed to several key methodological and clinical differences. Rahimzadeh et al.’s trial was limited to a small, homogeneous cohort with predominantly early-stage KOA (Kellgren–Lawrence grades 1–2), and only investigated the monotherapeutic effects of PRP and DPT. In contrast, Liao et al.’s meta-analysis incorporated a more heterogeneous and extensive patient population, including those with moderate to severe KOA, and assessed the combined effects of intra-articular injections with PT. The enhanced efficacy of DPT when used alongside PT may reflect a synergistic interaction that amplifies its therapeutic impact. Additionally, variations in injection protocols, frequency, PT modalities, and follow-up durations likely influenced the observed outcomes. In summary, PRP appears to offer greater benefits when used as a standalone treatment, particularly in early-stage KOA. However, DPT demonstrates superior clinical efficacy when integrated into a multimodal regimen with PT, especially for patients with more advanced disease. These findings underscore the importance of tailoring treatment strategies based on disease severity, therapeutic goals, and resource availability.

### DPT vs. PT

A randomized controlled trial (30) compared the efficacy of DPT and PT in improving symptoms of KOA in women. Sixty patients were randomly assigned to either the DPT group, which received intra-articular and peri-articular dextrose injections, or the PT group, which underwent treatments including hot packs, transcutaneous electrical nerve stimulation (TENS), and therapeutic ultrasound. Both groups showed significant improvements in pain, functionality, and muscle strength, measured by the VAS, WOMAC score, knee range of motion (ROM), and isokinetic strength. However, the DPT group exhibited superior results, particularly in pain reduction and muscle performance, at both 1 and 3 months post-treatment. These findings suggest that while both DPT and PT are effective, DPT offers greater improvements in managing KOA symptoms. A recent network meta-analysis on DTP and PT in KOA treatment shows that combined DTP and PT provides superior outcomes compared to monotherapy. DTP with PT is especially effective in reducing pain and improving global function. The combined approach showed a higher reduction in pain scores (SMD = -2.54) and greater restoration of global function (SMD = 2.28). This method outperforms other therapies and highlights the significance of combining regenerative and rehabilitative treatments for better patient outcomes (28).

### Intra-articular DPT vs peri-articular DPT

An increasing evidence indicates the different injection site of DPT yields different efficacy in treating KOA. A randomized controlled trial (31) compared the effectiveness of intra-articular (IG), peri-articular perineural injection (PG), and a combination of both (IG+ PG) in treating KOA. The results demonstrated that all three treatments significantly improved pain, functionality, and pressure pain threshold (PPT) when compared to baseline measurements. However, the combination therapy (IG + PG) showed superior outcomes, with significantly lower VAS and WOMAC scores at 4 and 8 weeks post-treatment, compared to either IG or PG alone. Additionally, PPT values were higher in the PG and IG + PG groups, indicating better alleviation of nerve sensitization. These findings suggest that ultrasound-guided combination therapy may offer a more effective and faster pain-relieving solution for KOA patients compared to single treatment modalities. On the contrary, there are evidence showing there are no differences regarding DPT injection site. A randomized controlled trial by Farpour et al. (32) evaluated the effectiveness of DPT, specifically comparing intra-articular and peri-articular injections for KOA treatment. Both methods resulted in significant improvements in pain relief and joint function, as measured by WOMAC, VAS, and OKS scores. No significant differences were found between the two injection techniques in terms of overall effectiveness. The study concluded that both intra-articular and peri-articular DPT are cost-effective and non-invasive treatment options for managing KOA symptoms. Similarly, a most recent double-blind, randomized clinical trial (33) assessed the analgesic efficacy of peri-articular DPT in patients with grade 2–3 bilateral knee OA. Twenty-six patients received DPT injections at acupuncture sites on one knee and non-acupuncture peri-articular points on the contralateral knee. Over a two-month follow-up, both groups exhibited significant improvements in pain, stiffness, and physical function, with no statistically significant difference between injection sites. These results suggest that the therapeutic benefits of DPT are independent of precise injection location, highlighting the technique’s robustness. The findings align with previous studies and support the use of peri-articular DPT as a safe, cost-effective intervention for KOA management. Despite limitations such as small sample size and short follow-up, the study reinforces prolotherapy’s clinical value and encourages further research into its mechanistic pathways.

## Limitations and challenges in DPT research

Research on DPT for KOA has shown promising potential; however, several methodological and practical limitations hinder the strength and generalizability of current evidence. One major concern is the substantial variability in study designs, particularly in DPT protocols. Differences in dextrose concentration, injection frequency, and the use of adjunctive treatments complicate direct comparisons. For example, while some studies employ combined intra-articular and periarticular injections (31, 32), others utilize a single approach (22, 34), reducing protocol consistency and limiting reproducibility. Similarly, the lack of standardization in outcome measures poses a challenge for data synthesis, as diverse tools—such as the WOMAC (24, 29), VAS (27), and the Knee Pain Scale (KPS) (15)—are inconsistently applied across trials. Risk of bias further compromises the validity of existing findings. Many studies suffer from small sample sizes (34–37), increasing the likelihood of underpowered analyses and potential Type II errors. The frequent absence of blinding (38) introduces performance and detection biases, particularly in studies reliant on subjective endpoints like pain. Additional heterogeneity arises from variations in OA severity across participants, often classified using different Kellgren-Lawrence radiographic grades (30, 33, 39), and from inconsistent reporting of outcome measures (15). Several trials have been rated as having high risk of bias, especially in performance and detection domains (17), which diminishes confidence in their conclusions. Another significant limitation is the short duration of follow-up in most DPT studies. The majority of trials assess outcomes within 3 to 6 months post-intervention (35, 40, 41), providing limited insight into the durability of treatment effects or long-term safety. This temporal gap highlights the need for extended observational periods to evaluate sustained clinical benefit and potential delayed adverse events. To support clinical decision-making, key practical considerations—including regulatory status, cost, safety, and contraindications—are summarized in **Table 1**.

**Table 1:** Practical considerations for hypertonic dextrose prolotherapy (DPT) in knee osteoarthritis.

Category	Details
Regulatory Status	DPT is generally used off-label in most countries. It is not currently approved by the FDA or EMA as a standard OA treatment (42).
Guideline Inclusion	Major guidelines (e.g., ACR, OARSI, NICE) do not include DPT in their core recommendations due to insufficient high-quality evidence.
Cost-Effectiveness	DPT is relatively low-cost compared to PRP or HA injections. Some studies suggest high patient compliance and lower dropout rates (23), making it a potentially cost-effective option, particularly in resource-limited settings.
Common Adverse Events	Mild post-injection pain, stiffness, or local swelling; generally self-limited. Serious adverse events are rare across clinical trials (23, 24).
Potential Contraindications	Active joint or systemic infection, uncontrolled diabetes, coagulopathy, or allergy to injectate components. Relative contraindications include acute gout, acute fracture, and anticoagulant therapy. Caution in patients with poor wound healing (43).

## Future research directions

Future research should address the biological mechanisms underlying DPT in KOA at the molecular and cellular levels. Elucidating the roles of cytokines, growth factors, and immune modulators involved in the healing response could enhance our understanding of its therapeutic action. Additionally, large-scale, multicenter randomized controlled trials with standardized protocols—such as dextrose concentration, injection frequency, and combination with adjunctive therapies like physical therapy—are essential to establish consistency across studies. Long-term follow-up is necessary to assess sustained efficacy and potential adverse effects. Moreover, studies exploring patient-specific variables such as age, KOA severity, and comorbidities can contribute to personalized treatment strategies. Finally, comparative effectiveness research using network meta-analysis could identify optimal treatment combinations for diverse clinical profiles.

## Conclusion

Prolotherapy represents a promising regenerative approach to treating KOA by stimulating natural tissue repair and improving joint function. Clinical evidence supports its effectiveness in reducing pain and enhancing mobility, often providing superior results compared to traditional therapies such as HA and corticosteroid injections. However, variability in treatment protocols and study designs, coupled with methodological biases, presents challenges to its broader acceptance. Standardized treatment protocols, more robust research methodologies, and longer-term studies are critical to fully understanding DPT’s potential and establishing it as a mainstream treatment option for KOA. Given the limitations of current therapies, DPT offers a valuable alternative, particularly for patients seeking non-invasive options to manage KOA symptoms.
